# Supplementary material for: Regulation of RNA granule dynamics by phosphorylation of serine-rich, intrinsically disordered proteins in C. elegans
Source: eLife. 2014 Dec 23;3:e04591. doi: 10.7554/eLife.04591 (PMC4296509; doi:10.7554/eLife.04591)
Supplement: Figure 2—source data 1. — DNA was extracted and sequenced from 111 colonies grown on –Trp–Leu–Ura–His plates. RNAi feeding vectors for each candidate were obtained from the Ahringer or OpenBiosystems RNAi banks, or if unavailable were PCR amplified from genomic DNA and cloned into pL4440. *meg-3(RNAi) also knocks out meg-4 and vice-versa. + in the pptr-1 suppression column means that the RNAi treatment restores P granules in P blastomeres. Pleiotropic indicates a P granule phenotype accompanied by additional cellular defects. Entries in red are the MEG proteins, which uniquely affect P granules in the zygote stage (meg-3 and meg-4) or suppress the pptr-1 phenotype (meg-1) without inducing other phenotypes. Other phenotypes were as follows: emb = embryonic lethal, dpy = dumpy, egl = egg laying defect. DOI: http://dx.doi.org/10.7554/eLife.04591.008 [file elife04591s001.docx]

| **ID** | **gene** | **Description** | **# of yeast colonies** | **P granule phenotype in WT** | **Suppression of the P granule phenotype in *pptr-1 (tm3103)*** | **Other phenotype** |
| --- | --- | --- | --- | --- | --- | --- |
| C27H6.3 | *tofu-1* | 21U-RNA biogenesis | 44 | - | - | None |
| Y71H2AM.20 |  | Phosphatase activator | 14 | - | - | 3.8% emb |
| K02B9.1 | *meg-1* | P granule component and regulator | 10 | - | + | 63% sterile (24^o^C) |
| Y37D8A.9 | *mrg-1* | chromodomain protein; essential for germ cell survival | 5 | - | - | 100% sterile |
| M110.4 | *ifg-1* | eIF4G | 3 | Somatic granules | Somatic granules | Larval lethal |
| C36C9.1 | *meg-4* | Homology to *gei-12* | 3 | Mislocalized in zygote* | N.D. | None |
| K02A4.1 | *bcat-1* | branched-chain amino acid aminotransferase | 3 | - | - | None |
| C11E4.6 |  |  | 2 | - | + | None |
| T09E8.2 | *him-17* | required for recombination during meiosis | 2 | - | - | None |
| T24C4.6 | *zer-1* | ubiquitin ligase substrate recognition subunit | 2 | - | - | None |
| F32H2.3 | *spd-2* | centrosome maturation | 2 | Pleiotropic | Enhanced | 100% emb |
| ZK6.11 |  |  | 2 | - | - | None |
| Y48E1C.1 |  |  | 2 | - | + | None |
| F46B6.7 | *ztf-7* | small yeast colony; Zinc finger Transcription Factor family | 2 | - | - | None |
| C27A12.3 | *tag-146* | small yeast colony; zinc finger protein | 2 | - | - | None |
| F29G9.2 |  |  | 2 | - | - | None |
| T01H3.4 | *perm-1* | required for eggshell formation | 1 | Mislocalized to C and D | - | 100% emb |
| W05B2.5 | *col-93* | collagen; cannot distinguish from col-92 and col-94 | 1 | - | - | None |
| F18A1.3? | *lir-1?* | LIN-26-like zinc-finger protein | 1 | - | - | None |
| F56F3.1 | *ifet-1* | eIF4E transporter | 1 | Pleiotropic | + | 79% emb |
| F52D2.4 | *gei-12/meg-3* | novel protein; homology to C36C9.1 | 1 | Mislocalized in zygote* | + | None |
| C09D1.1 | *unc-89* | small yeast colony; required for A bands in striated muscle | 1 | - | - | None |
| C09G5.5 | *col-80* | small yeast colony; collagen | 1 | - | - | None |
| Y24D9A.1 | *ell-1* | small yeast colony; ELL transcription elongation factor | 1 | - | - | dpy or small |
| W03C9.7 | *mex-1* | small yeast colony; P granule component and regulator | 1 | Mislocalized  after P_0_ | - | 98.8% emb |
| F26D10.3 | *hsp-1* | small yeast colony; HSP-70 | 1 | - | - | P0 mothers egl |
| C28H8.12 | *dnc-2* | small yeast colony; member of the dynamitin family | 1 | Pleiotropic | Pleiotropic | 98% emb |
| Y32H12A.5 | *paqr-2* | small yeast colony; Progestin and AdipoQ Receptor family | 1 | - | - | 6% emb |
| C55B7.4 | *acdh-1* | small yeast colony; also contained *meg-1* clone | 1 | - | - | None |
| F59D8.1 | *vit-3* | small yeast colony; also contained *meg-1* clone | 1 | - | - | 1.4% emb |
| Y39G10AR.7 |  | small yeast colony; also contained *meg-1* clone | 1 | - | - | 4.4% emb |
| K03A1.6 | *his-38* | small yeast colony; also contained *meg-1* clone | 1 | - | - | 6.6% emb |

Figure 2 – source data 1. Candidates from yeast two-hybrid screen

DNA was extracted and sequenced from 111 colonies grown on –Trp –Leu –Ura –His plates. RNAi feeding vectors for each candidate were obtained from the Ahringer or OpenBiosystems RNAi banks, or if unavailable, were PCR amplified from genomic DNA and cloned into pL4440. **meg-3(RNAi)* also knocks out *meg-4* and vice-versa.. + in the *pptr-1* suppression column means that the RNAi treatment restores P granules in P blastomeres. Pleiotropic indicates a P granule phenotype accompanied by additional cellular defects. Entries in red are the MEG proteins, which uniquely affect P granules in the zygote stage (*meg-3* and *meg-4*) or suppress the *pptr-1* phenotype (*meg-1*) without inducing other phenotypes. Other phenotypes were as follows: emb=embryonic lethal, dpy=dumpy, egl=egg laying defect.
